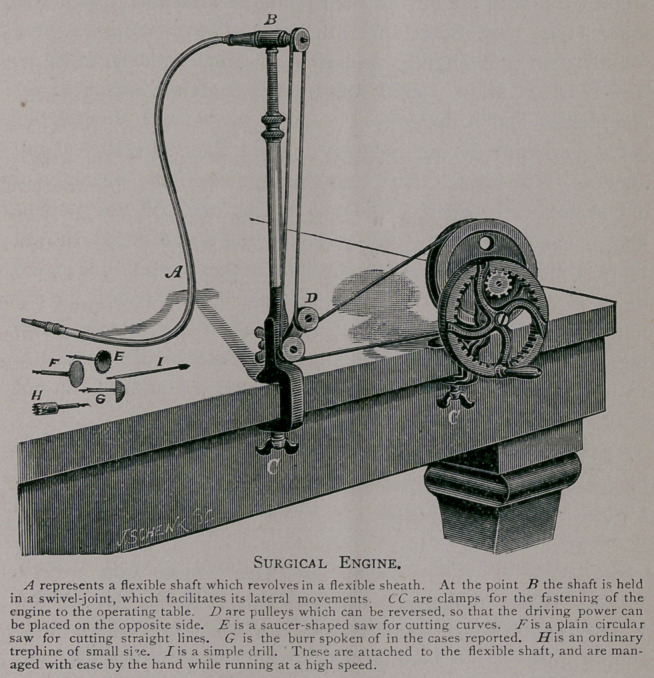# Injuries to the Cranial Bones—Trephining

**Published:** 1880-06

**Authors:** S. G. Dorr

**Affiliations:** Police Surgeon


### CLINICAL REPORTS.

---

### INJURIES TO THE CRANIAL BONES.—TREPHINING.

### REPORTS OF TWO CASES TREATED BY ELEVATING THE DEPRESSED BONE.

REPORTED BY S. G. DORR, M. D., POLICE SURGEON.

When to trephine, and when not to trephine, in fractures of the skull has always been a poorly defined subject. From time to time surgeons have advanced a step in the direction of more frequent operations, and to-day the only debatable ground is where there is a fracture, with depression and with an external wound, but without insensibility, coma or delirium shall the surgeon interfere with some operative procedure or not?

It seems strange that after all the advancement in medical science, this question should yet remain without a positive and never-varying affirmative answer. I think this question should be, at this time, answered affirmatively by every physician, and the debatable ground to exist only where there is fracture without external wound and without depression, unless the patient is quite young, and without insensibility, coma or delirium.

In looking over such records of cases, which have come to my knowledge, and by dividing them into three classes, I find some strange and startling results. The first class includes fractures of both tables, without any known depression; of this class the death rate was 64.6 per cent.; the second class, fractures of both tables, with known depression; of this class the death-rate was 35.8 per cent.; the third class of fractures, with depression of bone, with coma, delirium or insensibility, with formal trephining, and of this class 56, 6 per cent. died. It is not necessary to draw any extended deductions from these surprising results. All that I wish to occupy your valuable space in the JOURNAL is to deal, if possible, with some practical points which will benefit your readers and their patients. Of the third class I will not speak. They are not included in our proposition as first stated, that is, they do not occupy debatable ground. Of the second class there are fewer deaths than of either of the other two, and this is the class which justly occupies the present debatable ground. Some surgeons say, do not operate; others, it is best to trephine.

But all do agree upon one point, and that is if the broken parts are loose and easily to be gotton out, it is better to remove them. So that whether to trephine or not, in cases of the second, all depends upon the ability and ease of removal of the fractured parts. Hence, if by any means these fractured parts can always be removed with ease and without danger or difficulty, then always are those of the second class to be trephined and the question advanced, leaving those only of the first class to occupy the debatable ground. I will report two cases of the second class, the mode of operation and the results.

Late in the afternoon of April 17th, 1880, Geo. C., aged 21, presented himself at my office, with a depressed fracture, two and one-quarter inches long by three-eighths wide in the frontal bone extending from above the right eye directly upwards. This fracture had been produced by the bursting of a one-half inch thick emery wheel, worn somewhat thinner on the edge while running at a high speed.

The flesh and hair were driven in and held fast by the fractured bones, which were not broken loose, from the internal table, but had split lower, forming a long tongue, which had sufficient strength to hold very fast every thing that had been

SURGICAL ENGINE.

driven into its grasp. Here was a depressed fracture with no symptoms of compression, and the fractured parts well attached to the internal table; with the ordinary trephine very difficult to remove, but with the surgical engine there would be no difficulty whatever, and I determined to remove them and accordingly called in Drs. Van Peyma and Pettit, to assist in the operation.

Chloroform was administered to but a slight extent, and with an assistant to turn the crank of the instrument (which is so constructed as to give motion to burrs, drills, saws, &c., by means of a flexible shaft), I began burring off the overlapping edges of the outer table. As soon as the outer opening was enlarged a little over one-eighth of an inch, all the way round, and pieces slightly cut with the burr at their points of attachment with the inner table, they came out with ease. The membranes were found to be but slightly injured by a small spicula of bone. The external scalp wound was pushed together, and held by compresses and bandages. The pulse was 80 and showed but slight variation during recovery. Cold water dressings were applied to the head, comp. scammony powders and bromide of potassium were ordered. The following day only the patient kept his bed. In thirty-six hours after the operation he dressed himself, and from that time was about the house, and in ten days went to his work in the mill again, the wound having united without discharging hardly sufficient to stain the dressings. He says he suffered no more pain than he has frequently with headache. Now, thirty days after the operation, I can not see but that he has completely recovered.

Second Case: The evening of April 18, 1880, I was telephoned by Captain Yox, of the Eighth Precinct, to examine a saloon-keeper, who had been struck on the head with a mallet several hours previously.

The patient, a Frenchman, aged about 30, had a scalp wound on the right-parietal prominence; with my finger in the wound I could feel the edge of the fracture, and also about three-eighths of an inch deeper down, or depressed, a flat plate of bone, which was more or less loose. The patient showed no symptoms of compression of the brain. With the assistance of Dr. Van Peyma, our patient was trephined while under the influence of chloroform. The opening in the skull was one and a half by one and a quarter inches, and oval. The piece which was depressed and detached throughout its entire circumference by the blow from the mallet was, as is usual in these fractures, much larger than the opening in the external plate. The simple and easy way to get out such a piece is to make the opening larger. A rapidly revolving burr, driven by the surgical engine, was brought to bear against the overlapping edges of the outer table, which cut round and removed every obstruction to the elevation of the depressed oval plate. It was then picked out, showing below some small clots of blood, but no injury to the membranes. The scalp was pushed together, and the same treatment observed as in the first case. The next day the patient showed very much mental sluggishness. The third day he complained of hearing double, as if two persons were speaking, one repeating after the other; also weakness of the left arm and leg. From this time he made rapid and complete recovery, the pulse being most of the time 70, falling during the second day to 60. It will be observed by this mode of operating that only the outer table is cut away, and is therefore safe and simple. It makes the removal of broken bones a very easy operation. It likewise removes all arguments against the removal of depressed bone without waiting for coma, delirium or insensibilty.

## Figures and Tables

**Figure f1:**